# Health literacy and recovery following a non-catastrophic road traffic injury

**DOI:** 10.1186/s12889-022-13707-7

**Published:** 2022-07-19

**Authors:** Bamini Gopinath, Jagnoor Jagnoor, Annette Kifley, Ilaria Pozzato, Ashley Craig, Ian D. Cameron

**Affiliations:** 1 John Walsh Centre for Rehabilitation Research, Sydney Medical School, Kolling Medical Research Institute, University of Sydney, Sydney, Australia; 2 Macquarie University Hearing, Department of Linguistics, Faculty of Medicine, Health and Human Sciences, The Australian Hearing Hub, 16 University Avenue, Macquarie University, Sydney, NSW 2109 Australia; 3 The George Institute for Global Health, University of New South Wales, Sydney, Australia

**Keywords:** Health literacy, Non-catastrophic injury, Recovery, Road traffic crash

## Abstract

**Background:**

Health literacy (HL) is rarely addressed in rehabilitation research and practice but can play a substantial role in the recovery process after an injury. We aimed to identify factors associated with low HL and its relationship with 6-month health outcomes in individuals recovering from a non-catastrophic road traffic injury.

**Methods:**

Four hundred ninety-three participants aged ≥17 years who had sustained a non-catastrophic injury in a land-transport crash, underwent a telephone-administered questionnaire. Information was obtained on socio-economic, pre-injury health and crash-related characteristics, and health outcomes (quality of life, pain related measures and psychological indices). Low HL was defined as scoring < 4 on either of the two scales of the Health Literacy Questionnaire that covered: ability to actively engage with healthcare providers (‘Engagement’ scale); and/or understanding health information well enough to know what to do (‘Understanding’ scale).

**Results:**

Of the 493, 16.9 and 18.7% scored < 4 on the ‘Understanding’ and ‘Engagement’ scale (i.e. had low HL), respectively. Factors that were associated with low HL as assessed by both scales were: having pre-injury disability and psychological conditions; lodging a third-party insurance claim; experiencing overwhelming/great perceived sense of danger/death during the crash; type of road user; low levels of social satisfaction; higher pain severity; pain catastrophizing; and psychological- and trauma-related distress. Low HL (assessed by both scales) was associated with poorer recovery outcomes over 6 months. In these longitudinal analyses, the strongest association was with disability (*p* < 0.0001), and other significant associations were higher levels of catastrophizing (*p* = 0.01), pain severity (*p* = 0.04), psychological- (*p* ≤ 0.02) and trauma-related distress (*p* = 0.003), lower quality of life (*p* ≤ 0.03) and physical functioning (*p* ≤ 0.01).

**Conclusions:**

A wide spectrum of factors including claim status, pre-injury and psychological measures were associated with low HL in injured individuals. Our findings suggest that targeting low HL could help improve recovery outcomes after non-catastrophic injury.

## Introduction

The incidence of non-catastrophic injuries sustained in a land-transport crash has increased in the last three decades [1]. These injuries are associated with considerable personal, social, and economic health burden in the longer term [2–4]. Hence, there is a critical need to identify comprehensively factors hindering recovery following these traffic-related injuries, so that active support and management can be provided in a timely manner to improve long-term recovery outcomes.

Appropriate comprehension of the injury, rehabilitation, and treatment instructions plays an integral role in a patient’s health management and recovery process after the injury [5]. Health literacy (HL) (defined as the ability to obtain, process, and understand health information needed to make appropriate decisions concerning healthcare, disease prevention and health promotion to maintain or improve quality of life during the life course) [6] is a well-recognized but rarely studies in injury and rehabilitation research. A US study showed that every 1 in 4 trauma patients had low HL [7], and that disparities in socioeconomic status existed in HL in trauma patients. Specifically, low socio-economic status, and Hispanic versus Caucasian ethnicity were both associated with low HL. Further, low HL was associated with poor understanding of injuries and treatment provided, leading to decreased adherence with discharge instructions and longer recovery time [7]. *Hahn* et al. [8] showed that among people with spinal cord injury, stroke, or traumatic brain injury; higher HL was significantly associated with better overall health. Additionally, a single study of individuals with spinal cord injuries indicated that lower HL was associated with poorer physical mobility [9].

To our best knowledge, there are no cohort studies that have examined HL in persons who sustained non-catastrophic injuries in a land-transport crash. This is a population at high risk of decreased understanding of factors that influence their health. Some suggest this is because of the unexpected psychological and physical trauma and stresses associated with the crash compared to other patients (e.g. elective surgeries) [7]. Therefore, the objectives of this epidemiological study were to: 1) Determine the frequency of low HL in individuals who sustained a non-catastrophic injury in a land-transport crash; 2) Assess the factors that were associated with low HL among injured persons; and 3) Establish the independent associations between low HL and health outcomes (quality of life, psychological indices, and pain-related measures), over a 6-month follow-up period.

## Methods

### Study design

Study participants aged ≥17 years who had experienced a land transport crash resulting in a physical injury diagnosed by a medical practitioner in New South Wales (NSW), Australia, were interviewed within 28 days of injury [10]. Specifically, participants were identified from various sources including hospital emergency departments, general practitioners, and the claims database of a government insurance regulator. If the study site was a hospital emergency department, research nurses at each hospital site screened the “First Net” emergency department database to identify potential participants. Inclusion criteria were: a) injury due to crash involving a motorized vehicle on land (public/private road/driveway/parking space or private/public land) in NSW; b) injury due to motor vehicle crash diagnosed by a medical practitioner, or registered health practitioner, within 28 days of the crash; and c) injured person is a driver or passenger, motorbike rider, pillion passenger, pedestrian or bicyclist. Exclusion criteria were: a) superficial injuries (i.e. minor soft tissue and skin injuries that do not require specific management other than assessment and initial treatment) or injury due to a crash involving trains or light rail that are not covered by the NSW compulsory third party (CTP) scheme; b) dementia or significant pre-existing cognitive impairment affecting ability to consent; and c) sustained severe injuries (i.e. severe traumatic brain injury, spinal cord injury, extensive burns or multiple amputations), as these injuries are principally covered by an alternative insurance scheme in NSW [10].

**Fig. 1:**
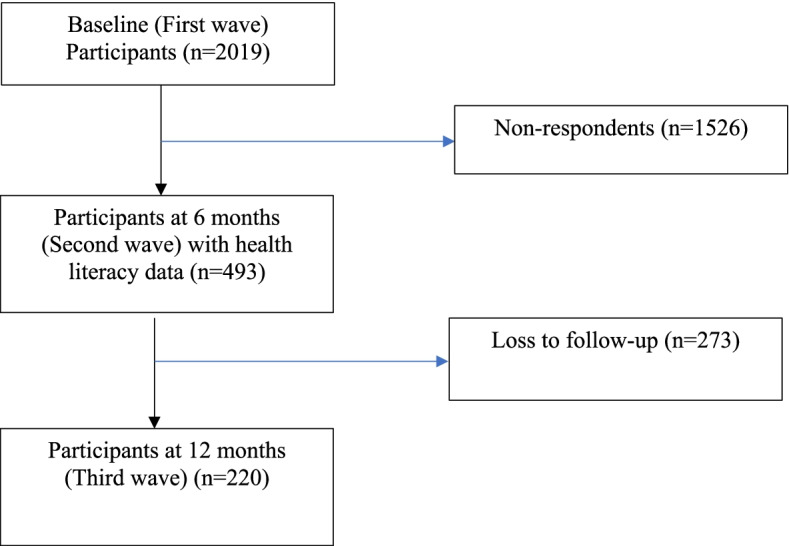
Study Flow

Once screened, potential participants were sent a letter that detailed the purpose of the study, what was involved and inviting them to participate in the study. Participants could opt-out of the study via telephone or through email. Participants who did not opt-out within one-week of the letter mail-out, were contacted by trained interviewers. Interviewers obtained informed consent by telephone and conducted the structured baseline interview [10]. A total of 2019 participants were recruited and surveyed at baseline (between August 2013 and December 2017; Fig. 1). Informed consent was obtained from all subjects and if subjects were under 18, informed consent was obtained from a parent and/or legal guardian. The study protocol and consent process were approved by a South Western Sydney Local Health District Human Research Ethics Committee. This study was conducted according to the principles expressed in the Declaration of Helsinki.

### Assessment of HL

Telephone-administered interviews assessed a suite of measures including HL. The HL profile of participants was assessed from the second study wave (Fig. 1) onwards (i.e. 6-month follow-up or near the end of 2015). Two scales from the Health Literacy Questionnaire (HLQ) [11] were used to assess levels of HL: 1) Understand health information well enough to know what to do (‘Understanding’ scale); and 2) Ability to actively engage with healthcare providers (‘Engagement’ scale). The two HL scales were chosen based on review of the available domains in the full HLQ scale. The two that were chosen were viewed as most relevant to people with recent trauma. The full scale was not used because of concern about informant burden.

Participants indicated how easy or difficult they believed it was to do a list of tasks within each of the ‘Understanding’ and ‘Engagement’ scales using a 5-point Likert response continuum ranging from: 1 (cannot do) to 5 (very easy). Scores for each scale are calculated for each respondent as the mean scores of the 5 items comprising the scale. Low HL was defined as scoring less than 4 on the ‘understanding’ and/or ‘engagement’ scale. This cut off score implied a response <=3 for at least one question on the subscale, and generally for more than one, hence, it represented self-report of at least some difficulty or problem on the subscale. Specifically, individuals with low scores on the ‘Understanding’ scale were characterized as: 1) Having problems understanding any written health information or instructions about treatments or medications; 2) Unable to read or write well enough to complete medical forms; and/or 3) Unable to follow accurately instructions from a health provider. Individuals with low scores on the ‘Engagement’ scale were characterized as: 1) Passive in their approach to healthcare or inactive i.e., they do not proactively seek or clarify information and advice and/or service options; 2) Unable to ask questions to get information or to clarify what they don’t understand; and/or 3) Feel unable to share concerns with a healthcare provider [11].

### Assessment of potential factors associated with HL

Interviews involved the collection of socio-demographic variables including, age, sex, education (university/tertiary or other), work status (paid work or other), country of birth, and marital status (married/defacto, divorced/widowed/separated, or never married).

Questions were asked on social satisfaction with the possible responses: 1) Completely or mostly satisfied; 2) Completely or mostly dissatisfied; or 3) Neither. Information on whether participants had lodged and/or were engaged in a CTP compensation claim following the accident was also collected. Presence of pre-injury comorbidities was determined by participants reporting whether they had any of the following: heart disease, stroke, arthritis, asthma, neurodegenerative diseases, visual or hearing impairments, chronic low back pain, and/or diabetes. Participants were also asked how many hours that they spent in hospital after the crash, and this was dichotomized as spending < 12 hours or ≥ 12 hours in hospital. The Abbreviated Injury Scale coding system was used to classify participants as: mild (1–3) and moderate (4–8) musculoskeletal injury groups based on the New Injury Severity Score [12]. Trained and experienced staff were used to code the reported injuries.

### Assessment of health outcomes

All recovery outcomes reported in this paper were measured over a 6-month period (i.e. at the third study wave; Fig. 1). The validated European Quality of Life-5 Dimensions (EQ-5D-3L) scale was administered and used to measure self-reported HRQoL pre-injury and post-injury [13]. The first part of the EQ-5D-3L had five dimensions: mobility, self-care, usual activities, pain/ discomfort and anxiety/ depression. Each dimension was divided into three levels: no problem, some problems and major problems. An individual’s health status can be described as a 5-digit numeral, calculated by combining the response to the five items (i.e. EQ-5D summary score). The second part is a 20-cm visual analogue scale (EQ VAS), which was modified slightly from the original version with a repetition of the question: ‘To help you say how good or bad your health state is, I have a scale in front of me (rather like a thermometer), on which the best health state you can imagine is marked 100 and the worst health state you can imagine is marked 0’ [13].

The Medical Outcomes Survey Short Form-12 (SF-12) was also administered and measures health-related quality of life [14]. Two component scores, the physical (SF-12 PCS) and mental component summaries (SF-12 MCS) were calculated directly as a weighted sum of individual items and a specified constant. Higher SF-12 MCS and PCS scores indicated better mental and physical wellbeing, respectively [15].

The Impact of Events Scale Revised (IES-R) is a validated 22-item self-report measure that assesses subjective distress associated with traumatic events [16]. Respondents were asked to indicate how much they were distressed during the past 7 days by their recent road crash experience. Items were rated on a 5-point scale ranging from 0 (‘not at all) to 4 (‘extremely’) with total scores ranging from 0 to 12, and higher scores indicated higher levels of distress. The Depression Anxiety Stress Scale-21 (DASS-21) is a validated and reliable 21-item scale that provides an overall assessment of general psychological distress or negative mood states; and domain scores for depressive mood, anxiety and perceptions of stress [17]. Participants were asked to complete 4-point Likert items (0–3) assessing the extent to which they have experienced psychological distress or negative mood states over the past week. Total scores ranged from 0 to 63 and were calculated by summing the scores for all 21 items [17]. The 12-item WHO Disability Assessment Schedule 2.0 (WHODAS short version) [18], including six domains: cognition, mobility, self-care, getting along, life activities and participation. A summary score ranging from 0 (‘no disability’) to 100 (‘full disability’) was obtained. The WHODAS reflects injury-related disability [18].

Mean pain severity was assessed using a 0 (‘no pain’) to 10 (‘worst pain imaginable’) numeric rating scale (NRS) to rate pain experienced over the past week. The Pain Catastrophizing Scale is a validated 13-item 6-point Likert scale with a range of 0–52 with scores of 34 or above indicating severely elevated pain-related catastrophic thinking styles [19]. However, due to a transposing error, a 6-point Likert scale (0-not at all to 5-all the time) was used rather than the usual 5-point scale, resulting in totals ranging between 0 and 65. These totals were rescaled so that the final score would lie on the published range of 0–52. Only the PCS total score data were presented.

### Statistical analysis

Statistical analyses were performed using SAS v9.4. Characteristics of participants with low HL were summarized using descriptive statistics and differences between groups on these variables were compared using t tests or χ^2^ tests where appropriate. Outcomes were modelled from baseline through to 6 months and 12 months. Health literacy was first measured at 6 months. There was a general view that health literacy could be regarded as a stable characteristic, therefore health literacy values were treated as fixed across time in the modelling. Covariates/adjustment factors were measured either preinjury or at baseline immediately after the injury. A linear mixed model analysis was used to determine differences between low and high HL groups for 6-month SF-12 and EQ-5D-3L scores, pain severity, pain catastrophizing, WHODAS, IES-R and DASS-21 scores while controlling for potential confounders. Consideration was given to the roles of the following factors using directed acyclic graphs: age, sex, preinjury health (comorbidities), preinjury disability (EQ5D summary scores), education, preinjury work, recruitment source, social satisfaction, preinjury history of anxiety or depression, crash role, perceived danger in crash, injury severity, hospital admission, pain severity, pain catastrophising, DASS21 and IESR scores, CTP claimant status. Beta coefficients with 95% confidence intervals and *p*-values are presented.

## Results

### Factors associated with low HL in injured persons

Of 2019 baseline participants, 493 completed HL questions at 6-month follow-up and so were included in subsequent analysis (Fig. 1). Of the 493, 16.9% (*n* = 83) and 18.7% (*n* = 92) scored < 4 on the ‘Understanding’ and ‘Engagement’ scale, respectively, and were classified as having low HL. Participants who completed the HL questions (respondents) versus those who did not (non-respondents) were older; more likely to be divorced/ widowed/ separated; admitted to the hospital > 12 hours; have pre-injury comorbidity and lodge a CTP claim, but less likely to be a cyclist and experience social dissatisfaction, at baseline or first study wave (Table 1). Mean scores (±SD) among study participants (*n* = 493) for the ‘Understanding’ and ‘Engagement’ scales were: 4.49 ± 0.70 and 4.40 ± 0.74, respectively.

**Table 1:** Comparison of characteristics of participants who did (respondents) versus those who did not (non-respondents) complete health literacy questions based on data collected at baseline or the first study wave

	**Non-respondents** **(*n*** **= 1526)**	**Respondents** **(*n*** **= 493)**	***P value***	***Subgroup*** ***p value***
**Age, years**				
17–24	307 (20.1)	80 (16.2)	0.002	0.055
25–44	646 (42.4)	184 (37.3)		0.047
45–59	377 (24.7)	134 (27.2)		0.3
60–69	109 (7.2)	52 (10.6)		0.02
70+	85 (5.6)	43 (8.7)		0.02
**Male gender**	989 (64.8)	316 (64.1)	0.77	
**Marital status**			< 0.0001	
Divorced, widowed or separated	132 (8.7)	72 (14.6)		0.0001
Married or defacto	742 (48.7)	272 (55.3)		0.01
Never married	651 (42.7)	148 (30.1)		< 0.0001
**Educational level**			0.15	
Primary or pre-primary	106 (7.0)	20 (4.1)		
Secondary	461 (30.3)	153 (31.0)		
Technical/ other further education	367 (24.1)	121 (24.5)		
Tertiary or university	1168 (76.5)	365 (74.0)		
**Pre-injury paid work or self-employment**	54 (65.1)	311 (76.0)	0.25	
**Injury severity score**				
12+	60 (3.9)	21 (4.3)		
9–11	96 (6.3)	34 (6.9)	0.22	
4–8	541 (35.5)	197 (40.0)		
1–3	829 (54.3)	241 (48.9)		
Pre-injury disability*	476 (31.2)	161 (32.8)	0.52	
**Pre-injury comorbidity**	663 (43.5)	272 (55.2)	< 0.0001	
**Lodging CTP claim**	349 (22.9)	155 (31.4)	0.0002	
**Pain severity ratings**	4.3 (2.7)	4.2 (2.7)	0.63	
**Total DASS-21 score**	13.0 (15.4)	12.1 (14.3)	0.25	
**Total IES-R scores**	3.7 (3.2)	3.5 (3.0)	0.14	
**Pain catastrophizing Scale scores**	14.1 (14.0)	13.1 (13.7)	0.18	
**Hospital stay > 12 hours**	750 (49.2)	275 (55.8)	0.01	
**Perceived sense of danger/death**			0.06	
Overwhelming	161 (10.8)	46 (9.5)		
Great	223 (14.9)	90 (18.5)		
Moderate	293 (19.6)	98 (20.1)		
Small	282 (18.9)	107 (22.0)		
None	534 (35.8)	146 (30.0)		
**Pre-injury psychological conditions**	357 (23.4)	133 (27.0)	0.11	
**Type of road user**				
Car driver	539 (35.3)	184 (37.5)		0.4
Car passenger	155 (10.2)	49 (10.0)	< 0.0001	0.9
Motorbike	445 (29.2)	183 (37.3)		0.0007
Cyclist	256 (16.8)	43 (8.8)		< 0.0001
Pedestrian/skateboard	131 (8.6)	32 (6.5)		0.14
**Social satisfaction**				
Dissatisfied	48 (3.2)	7 (1.4)		0.04
Neither	106 (7.0)	24 (4.9)	0.02	0.1
Satisfied	1370 (75.6)	389 (93.7)		0.01

Tables 2 and 3 show the factors that were associated with scoring < 4 on the ‘Understanding’ and ‘Engagement’ scale (i.e. low HL), respectively. Factors measured at 6 months and that characterized low HL as assessed by both scales included: lodging a CTP insurance claim; presence of pre-injury disability and psychological conditions; having an overwhelming perceived sense of danger/ death during the crash; not being a bicyclist; higher levels of pain severity and catastrophizing, and psychological and trauma-related distress post-injury. There were certain factors that were specifically associated with each of the HL sub-scales, that is, women versus men were more likely score < 4 on the ‘engagement’ scale, and participants who had only attained primary or pre-primary education were more likely to score < 4 on the ‘understanding’ scale.

**Table 2:** Factors associated with low health literacy (HL) as assessed by scoring < 4 on the ‘Understanding’ scale

**Baseline factors**	**Low HL** **(*n*** **= 83)**	**High HL** **(*n*** **= 409)**	***P value***	***Subgroup*** ***p value***
**Age, years**				
17–24	18 (21.7)	62 (15.2)	0.22	
25–44	26 (31.3)	158 (38.6)		
45–59	18 (21.7)	115 (28.1)		
60–69	12 (14.5)	40 (9.8)		
70+	9 (10.8)	34 (8.3)		
**Male gender**	51 (61.5)	265 (64.8)	0.56	
**Marital status**			0.74	
Divorced, widowed or separated	14 (17.1)	57 (13.9)		
Married or defacto	45 (54.9)	227 (55.5)		
Never married	23 (28.1)	125 (30.6)		
**Educational level**			0.001	
Primary or pre-primary	10 (12.1)	10 (2.4)		< 0.0001
Secondary	28 (33.7)	125 (30.6)		0.6
Technical/other further education	17 (20.5)	104 (25.4)		0.3
Tertiary or university	28 (33.7)	170 (41.6)		0.18
**Pre-injury paid work or self-employment**	54 (65.1)	311 (76.0)	0.04	
**Injury severity score**				
12+	7 (8.4)	14 (3.4)		
9–11	8 (9.6)	26 (6.4)	0.05	
4–8	25 (30.1)	171 (41.8)		
1–3	43 (51.8)	198 (48.4)		
**Pre-injury disability**^a^	39 (47.6)	122 (29.9)	0.002	
**Pre-injury comorbidity**	58 (69.9)	213 (52.1)	0.003	
**Lodging CTP claim**	40 (48.2)	114 (27.9)	0.0003	
**Pain severity ratings**	5.4 (2.6)	4.0 (2.6)	< 0.0001	
**Total DASS-21 score**	20.5 (17.9)	10.3 (12.8)	< 0.0001	
**Total IES-R scores**	5.4 (3.3)	3.1 (2.8)	< 0.0001	
**Pain catastrophizing Scale scores**	22.1 (16.0)	11.3 (12.4)	< 0.0001	
**Hospital stay > 12 hours**	50 (60.2)	225 (55.0)	0.38	
**Perceived sense of danger/death**			0.01	
Overwhelming	16 (19.8)	30 (7.4)		0.0005
Great	18 (22.2)	71 (17.5)		0.3
Moderate	13 (16.1)	85 (21.0)		0.3
Small	15 (18.5)	92 (22.7)		0.4
None	19 (23.5)	127 (31.4)		0.16
**Pre-injury psychological conditions**	31 (37.4)	101 (24.7)	0.02	
**Type of road user**				
Car driver	34 (41.0)	149 (36.6)		0.4
Car passenger	14 (16.9)	35 (8.6)	0.003	0.02
Motorbike	22 (26.5)	161 (39.6)		0.03
Cyclist	3 (3.6)	40 (9.8)		0.07
Pedestrian/skateboard	10 (12.1)	22 (5.4)		0.03
**Social satisfaction**				
Dissatisfied	3 (3.6)	4 (1.0)		0.06
Neither	8 (9.6)	16 (3.9)	0.01	0.03
Satisfied	72 (86.8)	389 (95.1)		0.004

*CTP* Compulsory Third-Party Insurance, *DASS-21* Depression Anxiety Stress Scale-21, *IES-R* Impact of Events Scale Revised

Data are presented as mean (SD) or n (%)

^a^As assessed by total European Quality of Life-5 Dimensions (EQ-5D-3L) scores

**Table 3:** Factors associated with low health literacy (HL) as assessed by scoring < 4 on the ‘Engagement’ scale

**Baseline factors**	**Low HL** (***n*** = 92)	High HL (***n*** = 401)	***P value***	***Subgroup*** ***p value***
**Age, years**				
17–24	18 (19.6)	62 (15.5)	0.61	
25–44	38 (41.3)	146 (36.4)		
45–59	22 (23.9)	112 (27.9)		
60–69	8 (8.7)	44 (11.0)		
70+	6 (6.5)	37 (9.2)		
**Male gender**	48 (52.2)	268 (66.8)	0.01	
**Marital status**			0.92	
Divorced, widowed or separated	13 (14.3)	59 (14.7)		
Married or defacto	52 (57.1)	220 (54.9)		
Never married	26 (28.6)	122 (30.4)		
**Educational level**			0.59	
Primary or pre-primary	5 (5.4)	15 (3.7)		
Secondary	32 (34.8)	121 (30.2)		
Technical/other further education	23 (25.0)	98 (24.4)		
Tertiary or university	32 (34.8)	167 (41.7)		
**Pre-injury paid work or self-employment**	62 (67.3)	303 (75.6)	0.11	
**Injury severity score**				
12+	3 (3.3)	18 (4.5)		
9–11	8 (8.7)	26 (6.5)	0.35	
4–8	30 (32.6)	167 (41.7)		
1–3	51 (55.4)	190 (47.4)		
**Pre-injury disability**^a^	45 (49.5)	116 (29)	0.0002	
**Pre-injury comorbidity**	56 (60.9)	216 (53.9)	0.22	
**Lodging CTP claim**	47 (51.1)	108 (26.9)	< 0.0001	
**Pain severity ratings**	5.3 (2.6)	4.0 (2.6)	< 0.0001	
**Total DASS-21 score**	21.5 (17.0)	9.9 (12.7)	< 0.0001	
**Total IES-R scores**	5.3 (3.2)	3.0 (2.8)	< 0.0001	
**Pain catastrophizing Scale scores**	21.3 (15.0)	11.3 (12.8)	< 0.0001	
**Hospital stay > 12 hours**	49 (53.3)	226 (56.4)	0.59	
**Perceived sense of danger/death**			0.04	
Overwhelming	16 (17.6)	30 (7.6)		0.003
Great	18 (19.8)	72 (18.2)		0.7
Moderate	18 (19.8)	80 (20.2)		0.9
Small	17 (18.7)	90 (22.7)		0.4
None	22 (24.2)	124 (31.3)		0.18
**Pre-injury psychological conditions**	37 (40.2)	96 (23.9)	0.002	
**Type of road user**				
Car driver	38 (41.3)	146 (36.6)		0.4
Car passenger	13 (14.1)	36 (9.0)	0.01	0.14
Motorbike	27 (29.4)	156 (39.1)		0.08
Cyclist	3 (3.3)	40 (10.0)		0.04
Pedestrian/skateboard	11 (12.0)	21 (5.3)		0.02
**Social satisfaction**				
Dissatisfied	3 (3.3)	4 (1.0)		
Neither	7 (7.6)	17 (4.2)	0.10	
Satisfied	82 (89.1)	380 (94.8)		

*CTP* Compulsory Third-Party Insurance, *DASS-21* Depression Anxiety Stress Scale-21, *IES-R* Impact of Events Scale Revised

Data are presented as mean (SD) or n (%)

^a^As assessed by total European Quality of Life-5 Dimensions (EQ-5D-3L) scores

### Associations between low HL and 6-month health outcomes

Tables 4 and 5 show multivariate-adjusted associations between low HL (scoring < 4 on the ‘Understanding’ and ‘Engagement’ scale), and health outcomes over a 6-month follow-up period. Low HL as assessed by both scales was significantly associated with poorer health-related quality of life and physical functioning (lower EQ-5D-3L and SF-12 PCS scores); and higher levels of pain severity, catastrophizing and psychological- and trauma-related distress (higher DASS-21 scores and IES-R scores), 6 months later. Further, those who scored < 4 on the ‘Engagement’ scale had significantly poorer mental wellbeing i.e. lower SF-12 MCS scores (Table 5).

**Table 4:** Temporal associations between low health literacy (score < 4 on ‘Understanding’ scale) and health outcomes in injured participants, assessed over a 6-month period

	**Low Health Literacy (based on ‘Understanding’ scale)**		
**Health Outcomes (each unit-increase)**^a^	**β (95% CI)**	***P*** value	**Effect size**
SF-12 PCS	−5.15 (−7.99, −2.32)	0.0004	0.5 SDs
EQ-5D-3L summary score	−0.10 (−0.17, 0.03)	0.004	0.13 SDs
WHODAS score	9.04 (4.53, 13.55)	< 0.0001	0.51 SDs
Pain severity ratings	0.68 (0.02, 1.34)	0.04	0.28 SDs
DASS-21 total score	4.17 (0.67, 7.66)	0.02	0.31 SDs
IESR total score	0.95 (0.32, 1.56)	0.003	0.34 SDs
Pain catastrophizing score	4.46 (1.27, 7.66)	0.01	0.36 SDs

*DASS-21* Depression Anxiety Stress Scale-21, *EQ-5D-3L* European Quality of Life-5 Dimensions, *IES-R* Impact of Events Scale Revised, *PCS* Physical Component Summary Score, *WHODAS* WHO Disability Assessment Schedule

^a^Adjusted for age, sex, education, social satisfaction, remoteness, pre-injury factors (anxiety/depression, disability, comorbidities and employment), type of road user, injury severity scores, hospital admission, pain severity, perceived danger, psychological factors (DASS-21, IESR, catastrophizing) and third-party insurance claim status

**Table 5:** Temporal associations between low health literacy (score < 4 on ‘Engagement’ scale) and health outcomes in injured participants, assessed over a 6-month period

	**Low Health Literacy (based on ‘Engagement’ scale)**		
**Health Outcomes (each unit-increase)**^a^	**β (95% CI)**	***P*** value	**Effect size**
SF-12 PCS	−3.77 (−6.64, − 0.89)	0.01	0.36 SDs
SF-12 MCS	−3.80 (−6.52, −1.07)	0.01	0.38 SDs
EQ-5D-3L summary score	−0.078 (− 0.15, 0.007)	0.03	0.1 SDs
WHODAS score	9.44 (4.92, 13.95)	< 0.0001	0.53 SDs
Pain severity ratings	0.71 (0.04, 1.37)	0.04	0.29 SDs
DASS-21 total score	7.37 (3.92, 10.82)	< 0.0001	0.55 SDs
IESR total score	0.96 (0.34, 1.58)	0.003	0.34 SDs
Pain catastrophizing score	4.47 (1.25, 7.68)	0.01	0.36 SDs

*DASS-21* Depression Anxiety Stress Scale-21, *EQ-5D-3L* European Quality of Life-5 Dimensions, *IES-R* Impact of Events Scale Revised, *MCS* Mental Component Summary Score, *PCS* Physical Component Summary Score, *WHODAS* WHO Disability Assessment Schedule

^a^Adjusted for age, sex, education, social satisfaction, remoteness, pre-injury factors (anxiety/depression, disability, comorbidities and employment), type of road user, injury severity scores, hospital admission, pain severity, perceived danger, psychological factors (DASS-21, IESR, catastrophizing) and third-party insurance claim status

## Discussion

This epidemiological study shows that close to one in five participants with a non-catastrophic injury had low HL. A wide range of correlates (sociodemographic, pre-injury, psychological and crash-related factors) were associated with low HL in this cohort of injured individuals. Low HL was associated with poorer recovery outcomes including higher levels of catastrophizing, disability, psychological distress and pain severity ratings; and lower quality of life and physical functioning over 6 months.

The prevalence of low HL is comparable to, albeit slightly lower, than rates observed in other studies which were in the magnitude of 14–40% [7, 9, 20]. These prior studies had surveyed persons who had sustained traumatic/ catastrophic injuries, that is, differing severity and type of injury, while ours included only persons with non-catastrophic injuries and this could account for the differences in observed rates. Other underlying reasons for observed differences could be variations in the scales used to assess HL; and the age, sex and ethnic group distribution of study participants across these studies. Nevertheless, our study findings provide unique insights and underscore the difficulties that persons with non-catastrophic injuries are likely to experience when accessing, using and attempting to understand injury and recovery information in the healthcare system. These challenges and difficulties could arise due to injured persons having to navigate complex and unfamiliar language; deal with inconsistent and incomplete injury and recovery information; and integrate information provided from numerous and diverse health professionals as well as other relevant groups (e.g. insurance companies and lawyers) [21].

We report on risk factors that could potentially identify individuals with low HL and these could be easily communicated to healthcare professionals routinely treating people with mild/ moderate injuries. Pre-injury disability and psychological conditions, as well as high levels of catastrophizing; psychological and trauma-related distress measured early post-injury were all independently associated with low HL. These findings indicate that the psychological and physical stresses experienced by the individual post-injury, could lead to decreased knowledge motivation and reduced understanding of health and injury information, and how the healthcare system worked [7]. Further, these stresses in injured persons could also lead to a lack of confidence to communicate their own values and preferences as well as advocacy skills to ensure quality of healthcare services delivered [21]. Moreover, engagement with the CTP insurance scheme is likely to compound the psychological and physical stresses that these individuals might experience after the crash. Indeed, prior research showed that lodging a claim and seeking compensation following a land-transport crash increases risk of psychological distress in claimants [22–24]. Prolonged exposure to the insurance scheme also increases the likelihood of participants coming into contact with system complexities which are known to be stressful [23] including; numerous assessments [25] and the overall adversarial nature of contacts with claims staff [26, 27]. Hence, these mechanisms could underlie the strong link between lodging a CTP insurance claim and low HL in our study.

Despite the growing recognition of health literacy as a barrier that affects individual health care and public health [28], there is limited research about its effect on recovery outcomes following non-catastrophic injuries. Our findings provide new knowledge that low HL is independently associated with range of poor health outcomes and incomplete recovery after 6 months in those with minor/ moderate injuries. These findings agree with prior cohort studies of individuals who sustained catastrophic injuries, where low HL was associated with a longer time to recovery [7] and greater physical health morbidity post-injury [9]. In our study, low HL appeared to influence 6-month recovery outcomes independent of the confounding influences of sociodemographic measures (e.g. age, sex and education), pre-injury factors, acute psychological factors, CTP claim status, and crash-related characteristics. These findings highlight the potential value of brief screening tools in identifying persons lacking HL skills; thereby, reducing their risk of poorer recovery in the longer term. The two scales that we used in the current study form part of a more comprehensive 9-item Health Literacy Questionnaire [11], and it is likely that other scales of this questionnaire could be incorporated as part of a screening tool that could provide a complete profile that captures the variety of health literacy needs in those who have sustained a non-catastrophic injury. This should be tested in larger cohort of injured participants followed up for a longer duration after the crash.

It has been suggested that better integration of health literacy, health equity, and patient-centred care initiatives [29] would help to shift the focus from the negative effects of low HL [30] to a positive model of how health literacy can be used to improve recovery outcomes. To this end, various evidence-based interventions have been proposed and examined to improve health literacy or patients’ comprehension in the context of other health conditions [31, 32]; and these could be of value in those who have sustained mild/ moderate injuries. Specifically, personalized written and verbal documentation of injuries, treatment/ rehabilitation plans and available services by hospital ED staff presented in plain language and in a variety of formats (online, print and in-person), would likely assist patients and their relatives to coordinate and integrate information once leaving the hospital and over the course of their recovery [20]. Moreover, interventions could be implemented that train injured persons to communicate in a way to increase their ability to obtain information, participate in their healthcare and receive person-centred care [21, 33].

Strengths of this study include its prospective design and the robust collection of data on a wide range of health outcomes and explanatory variables using reliable and validated instruments. However, our findings need to be interpreted with caution due to study caveats. First, we cannot disregard residual confounding from factors that were not measured or accounted for, such as hospitalization details (e.g. procedures undergone in hospital) and personality factors (e.g. self-efficacy, resilience). Second, we had self-reported measures of pre-injury characteristics (e.g. presence of disability and psychological conditions) and as a result several aspects of bias can arise which might have influenced observed associations. Third, we only administered questions to assess HL 6 months after the crash, which could have resulted in some participants to over- or under-estimate their level of HL and we cannot disregard the possibility that the level of HL might have improved somewhat or people could report more difficultly on these questions if they are experiencing psychological distress or encounter more difficult trauma or claim-related experiences after the crash. Fourth, there were significant differences between respondents and non-respondents in terms of e.g. age, type of road user, marital status, presence of pre-injury comorbidity, CTP claim status, and hospital admission. Therefore, we cannot disregard the possibility of selection bias influencing our observed associations, which limits the generalizability of our study findings. The data for non-respondents suggests that at a population level, the impact on recovery outcomes is likely smaller because of the differential drop-out of more people with e.g. less pre-injury comorbidity at baseline. This bias is likely to be compounded by the low follow-up rate (< 50%), as the reduction in participant numbers at follow-up could have underestimated some of the associations between low baseline HL and 6-month outcome measures. However, the directionality of the association is unlikely to be influenced by this bias, that is, the most likely direction for the relationship is that low health literacy is associated with poorer recovery outcomes as a result of sustaining a non-catastrophic injury. A reverse direction of effect (poor recovery outcomes due to sustaining a non-catastrophic injury leading to significantly lower health literacy levels) seems less likely.

Finally, we only administered two scales from the HLQ and each of the HLQ scales are designed to provide pertinent and unique information on different aspects of HL, therefore, by only administering two of the scales it is likely that we may have not comprehensively established the HL profile of injured persons.

## Conclusions

In summary, we found that nearly one in five injured persons had low HL. A wide spectrum of factors factors including claim status, pre-injury and psychological measures characterized low HL among injured persons. Low HL was associated with incomplete recovery and poorer health outcomes over a 6-month follow-up. Our findings, therefore, suggest that improvement in long-term recovery outcomes in persons who sustained non-catastrophic injuries could be achieved through addressing their knowledge and information needs, reducing the complexity of the HL environment, and improving patient-centred communication.

## Data Availability

The datasets used and/or analysed during the current study are available from the corresponding author on reasonable request.

## Abbreviations

CTP: Compulsory Third Party
DASS-21: Depression Anxiety Stress Scale-21
ED: Emergency Department
EQ-5D-3L: European Quality of Life-5 Dimensions
HL: Health Literacy
HLQ: Health Literacy Questionnaire
IES-R: Impact of Events Scale Revised
NRS: Numeric Rating Scale
NSW: New South Wales
SF-12: Survey Short Form-12
VAS: Visual Analogue Scale
WHODAS: WHO Disability Assessment Schedule
